# Comparison of Newly Diagnosed Ocular Hypertension and Open-Angle Glaucoma: Ocular Variables, Risk Factors, and Disease Severity

**DOI:** 10.1155/2012/757106

**Published:** 2011-08-18

**Authors:** Yvonne M. Buys, Paul Harasymowycz, Rania Gaspo, Kenneth Kwok, Cindy M. L. Hutnik, Pierre Blondeau, Catherine M. Birt, Robert L. G. Piemontesi, Lisa F. Gould, Mark R. Lesk, Iqbal K. Ahmed

**Affiliations:** ^1^Department of Ophthalmology, University of Toronto, 399 Bathurst Street, EW 6-405, Toronto, ON, M5T 2S8, Canada; ^2^Department of Ophthalmology, Toronto Western Hosptial, 399 Bathurst Street, EW 6-405, Toronto, ON, M5T 2S8, Canada; ^3^Department of Ophthalmology, University of Montreal, Montréal, QC, H3T 1J4, Canada; ^4^Medical Advisor - Oncology, Pfizer Canada Inc., Kirkland, QC, H3C 3J7, Canada; ^5^Biostatistics, Pfizer Inc., New York, NY 10017, USA; ^6^Ivey Eye Institute, St. Joseph's Health Care London, London, ON, N6G 1J1, Canada; ^7^Department of Ophthalmology, University of Sherbrooke, Sherbrooke, QC, J1K 2R1, Canada; ^8^Department of Ophthalmology, Naniamo, British Columbia, V9R 5B6, Canada; ^9^The Winnipeg Clinic, Winnipeg, MB, R3C ON2, Canada; ^10^Department of Ophthalmology, Hopital Maisonneuve-Rosemont, Montréal, QC, H1T 2M4, Canada

## Abstract

*Purpose*. To describe the distribution of ocular variables, risk factors, and disease severity in newly diagnosed ocular hypertension (OH) or open-angle glaucoma (OAG). *Methods*. Eligible subjects underwent a complete history and examination. Adjusted odds ratios (ORs) and 95% confidence intervals (CIs) obtained from multiple logistic regression models were used to compare OAG to OH and advanced to early/moderate OAG. *Results*. 405 subjects were enrolled: 292 (72.1%) with OAG and 113 (27.9%) with OH. 51.7% had early, 27.1% moderate, and 20.9% advanced OAG. The OR for OAG versus OH was 8.19 (*P* < 0.0001) for disc notch, 5.36 (*P* < 0.0001) for abnormal visual field, 1.45 (*P* = 0.001) for worsening mean deviation, 1.91 (*P* < 0.0001) for increased cupping, 1.03 for increased age (*P* = 0.030), and 0.36 (*P* = 0.010) for smoking. *Conclusions*. Increased age was a risk for OAG, and smoking decreased the risk of OAG compared to OH. Almost half of the OAG subjects had moderate/advanced disease at diagnosis.

## 1. Introduction

Understanding disease risk factors is important for diagnosis and treatment. Several glaucoma risk factors are known; however, the literature is occasionally conflicting, suggesting that risk factors may vary depending on glaucoma type and stage [1]. Presently, risk factors with strongest evidence for conversion of ocular hypertension (OH) to glaucoma, or presence and progression of open-angle glaucoma (OAG), include older age, thinner central corneal thickness (CCT), greater cup-to-disc ratio (C/D ratio), and higher intraocular pressure (IOP). 

Other potential risk factors for the development or progression of OAG include gender [2–4], race [5], body mass index (BMI) [6], myopia [7–11], bilateral disease [12], family history of glaucoma [7, 13–16], hypertension [7, 17–20], hypotension [21–23], smoking [24], thyroid disease [25, 26], migraine [4, 27], Raynaud's disease [28], concomitant medications (including corticosteroids, alpha-blockers, statins) [29–31], asymmetric [32] and diurnal variation in IOP [33], sleep apnea [34], phakic status [35], optic disc size [36], disc hemorrhage [37], pseudoexfoliation [8, 37], and pigment dispersion [38].

The varying results of studies on glaucoma risk factors require further research. We describe the presence and distribution of previously published glaucoma risk factors in patients newly diagnosed with OH or OAG comparing these two groups and analyze the distribution of risk factors in relation to OAG disease severity.

## 2. Materials and Methods

This was a multicentre, prospective, noninterventional, cross-sectional study registered with http://ClinicalTrials.gov NCT00334750 conducted at 18 Canadian centers in accordance with the Declaration of Helsinki and approved by the ethics committee of each center. 

Inclusion criteria included minimum age of 18 years, diagnosed at or within 3 months of the study visit with OH (at least one measurement of IOP ≥ 24 mm Hg and no evidence of glaucomatous damage) or OAG (open angle on gonioscopy and any two of the following: IOP ≥ 21 mmHg, glaucomatous optic neuropathy, and glaucomatous visual field defect), and willing and able to comply with study procedures, and provide written informed consent. Exclusion criteria included subjects previously or currently receiving ocular hypotensive therapy, a history of ocular surgery (except cataract surgery), or ocular trauma.

The study consisted of one visit for each subject; however, some assessments were allowed within one month of enrolment. The following information was collected on standardized data collection sheets: demographics, medical history, ocular family history, and complete ophthalmic examination including visual fields. Ocular data included refraction, best corrected visual acuity, pupil responses, slit-lamp biomicroscopy, and gonioscopy. Diurnal IOP (08:00, 10:00, 12:00, 14:00, and 16:00) was measured with a calibrated Goldmann applanation tonometer. CCT was measured ultrasonically with 3 readings per eye averaged. Dilated fundus examination was performed to assess disc size, C/D ratio, and presence of disc hemorrhage, peripapillary atrophy, and nerve fiber layer defect (NFLD). Optic disc size was classified based on comparing disc area with the target light of a direct ophthalmoscope's smallest aperture. If the disc fits the target it was classified “normal,” if smaller it was classified “small,” and if larger, it was classified “large.” Visual fields were performed using the Humphrey Field Analyzer 24-2 Swedish Interactive Thresholding Algorithm (SITA) Fast (Carl Zeiss Meditec, Dublin, Calif, USA) and included the Glaucoma Hemifield Test (GHT) analysis. Information obtained included mean deviation (MD), pattern standard deviation (PSD), and any abnormalities. 

Classification as OH or OAG was determined by the study site. Severity of glaucoma was based on visual field and optic disc examination: advanced (C/D ratio ≥ 0.9 or MD <  −12.0 dB or VF defect within 10° of fixation), moderate (C/D ratio 0.65–0.9 or MD −5.0 to −12.0 dB, no VF defect within 10° of fixation), and early (C/D ratio ≤0.65 or MD > −5.0 dB, no VF defect within 10° of fixation) [39].

### 2.1. Statistical Analysis

All analyses were based on data from a single eye per subject. If both eyes qualified, the eye with the worst disease was included; if equal, the right eye was designated.

Descriptive statistics of demographic characteristics and other potential risk factors for the two conditions (OAG and OH) were provided. To compare differences between the two, mean difference (and SD), 95% CI of the mean difference and *P*-value were calculated for continuous variables, and odds ratio (OR), 95% CI of odds ratio, and *P*-value for binary variables.

To assess which risk factors and/or ocular variables were associated with OAG versus OH, adjusted ORs, along with 95% CIs, were obtained from multiple logistic regression models with diagnosis (OAG versus OH) as the response variable and risk factors and/or ocular variables as independent variables. Correlation matrices checked for colinearity between potential risk factors and/or ocular variables within each of 3 classes of variables; highly correlated variables may not have been jointly considered within a model. Potential risk factors were selected based on a stepwise procedure using entry fixed at 5% and stay fixed at 10%. OR and 95% CIs were presented for the selected factors in the final model (also called reduced model). 

Additionally, to assess which risk factors and/or ocular variables were associated with severity of OAG, ordinal logistic regression (proportional odds model) was performed, with severity of disease (early, moderate, and advanced) as a response variable and risk factors and/or ocular variables as independent variables. Statistical analysis was performed using SAS version 9.

## 3. Results

410 subjects were enrolled. Information regarding diagnosis was available for 405 subjects who were analyzed. Overall, 404 (98.5%) subjects completed the study and 6 (1.5%) subjects discontinued.

292 (72.1%) of 405 subjects were diagnosed with OAG and 113 (27.9%) with OH. In OAG most had primary open-angle glaucoma (POAG, 202/292, 69.2%). Other diagnoses included normal tension glaucoma (65/292, 22.3%), pseudoexfoliation (16/292, 5.5%), and pigmentary glaucoma (9/292, 3.1%). 151 (51.7%) of 292 subjects had early OAG at diagnosis; a similar percent had moderate or advanced OAG: 79/292 (27.1%) and 61/292 (20.9%), respectively. Overall 60.5% of subjects (245/405) had symmetrical disease compared with 39.5% (160/405) with asymmetrical disease. A higher percentage of subjects with OH had symmetrical findings (73.5%) than subjects with OAG (55.5%). 

Table 1 summarizes the demographic characteristics. Subjects with newly diagnosed OAG were significantly older than those with OH (63.0 ± 13.0 years for OAG and 56.5 ± 12.6 for OH, *P* < 0.0001). The distribution of male (145 OAG versus 59 OH) and female (147 OAG versus 54 OH) subjects was similar within the groups. The majority of subjects were Caucasian (348/405; 85.9%) and the proportion of Caucasian subjects was greater in OH, *P* = 0.013. Mean weight and BMI were both greater in OH, *P* = 0.004 and 0.001, respectively.

Table 2 summarizes the ocular examination results and Table 3 the optic nerve assessment. The OAG subjects had lower mean IOP, thinner CCT, and greater degree of myopia than OH subjects. 6.2% of the OAG subjects had a relative afferent pupillary defect (RAPD) compared to none in OH subjects, *P* = 0.005. For the optic nerve characteristics evaluated, more OAG subjects had abnormalities than OH. The mean C/D ratio was 0.7 ± 0.2 in OAG and 0.4 ± 0.2 in OH, *P* < 0.0001. There were more large discs in OAG (13.7%) compared to OH (6.2%), *P* = 0.035. 

Table 4 summarizes the VF results. For each measurement, the median was approximately double or higher for the OAG subjects; however, the 95% CIs were significantly different for only MD, PSD, and false negative rate, and there was a trend towards significance for fixation losses with *P* = 0.058. 

The majority of subjects in both OAG and OH groups had no family history of glaucoma (62.0%) or elevated IOP (87.9%). Subjects with OH reported less frequent family history of blindness (3.5%) than subjects with OAG (11.0%), *P* = 0.019. Overall, 45.1% of subjects with OH reported a family history of glaucoma, elevated IOP, or blindness compared with 42.1% of subjects with OAG.

Table 5 summarizes glaucoma risk factors by subjects' medical histories; past or current histories were classified as “yes.” The most frequently reported medical histories for the OAG and OH groups, respectively, were vascular dysfunction (43.8% and 32.7%), smoking (29.5% and 46.9%), and hypercholesterolemia (31.8% and 27.4%). There were no significant differences between the groups for all medical conditions evaluated except vascular dysfunction and smoking with *P*-values of 0.042 and 0.001, respectively. 

The medication most frequently taken was statins, reported by 83 (28.4%; 95% CI: 23.32–33.97) of 292 OAG subjects and 23 (20.4%; 95% CI: 13.49–29.20) of 113 OH subjects. There were no significant differences between OAG and OH subjects for any concomitant medication including statins, corticosteroids, or alpha blockers.

**Figure 1:**
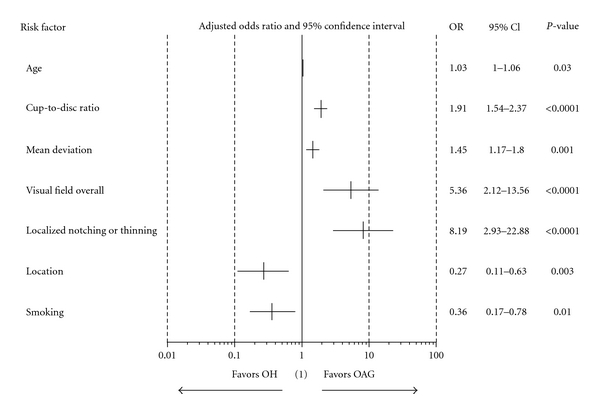
Association of primary diagnosis versus risk factors (reduced model). (i) Primary diagnosis: OAG versus OH. (ii) Age, cup-to-disc ratio, and mean deviation are continuous variables, and the others are categorical. Age was estimated in every one-year increment, cup-to-disc ratio in every 0.1-unit increment, and mean deviation in every 1 dB unit of worsening. Categorical variables were estimated as follows: visual field overall (abnormal versus normal, localized notching/thinning (Yes versus No), location (Quebec/Atlantic versus Ontario), smoking (Yes versus No). (iii) Odds ratio, 95% confidence interval. (1) OR = 1 implies a specific risk factor is equally likely in both OAG and OH groups, OR >1 implies more likely in OAG, and OR < 1 implies more likely in OH.

Figure 1 shows which risk factors and ocular variables were significantly associated with diagnosis (OAG versus OH) in the reduced model. The predicted odds ratio of OAG for localized notch or thinning of neuroretinal rim was 8.19; subjects with these factors were 8.19 times more likely to have OAG than OH (95% CI: 2.93–22.88; *P* < 0.0001). Subjects who smoked had 0.36 times the odds of having OAG than OH (95% CI: 0.17–0.78; *P* = 0.010). Additional significant risk factors were age (OR 1.03; 95% CI 1.00–1.06; *P* = 0.030) per year increase, C/D ratio (OR 1.91; 95% CI: 1.54–2.37; *P* < 0.0001) per 0.1 unit increase, MD (OR 1.45; 95% CI 1.17–1.80; *P* = 0.001) per 1 dB decrease, and abnormal VF (OR 5.36; 95% CI: 2.12–13.56; *P* < 0.0001).

**Figure 2:**
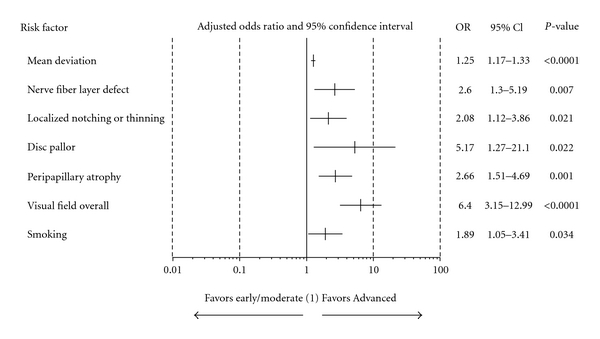
Association of severity of OAG versus risk factors (reduced model). (i) severity of OAG. Advanced disease versus early or moderate disease. (ii) Mean deviation (MD) is a continuous variable, and the others are categorical. MD was estimated in every 1 dB unit of worsening. Categorical variables were estimated as follows: nerve fiber layer defect, localized notching/thinning, disc pallor, peripapillary atrophy, smoking (Yes versus No) and visual field overall (abnormal versus normal). (1) OR = 1 implies a specific risk factor is equally likely in both severity levels, OR > 1 implies more likely in advanced, and OR < 1 implies more likely in early/moderate.

Figure 2 shows which factors were associated with advanced versus early or moderate OAG. Subjects with diffuse or localized areas of pallor had over 5 times the odds as subjects without pallor of having advanced OAG (95% CI: 1.27–21.10; *P* = 0.022) at diagnosis. Additional significant factors included worse MD (OR 1.25; 95% CI: 1.17–1.33; *P* < 0.0001), NFLD (OR 2.60; 95% CI: 1.30–5.19, *P* = 0.007), localized notch or rim thinning (OR 2.08; 95% CI: 1.12–3.86; *P* = 0.021), presence of peripapillary atrophy (OR 2.66; 95% CI: 1.51–4.69; *P* = 0.001), abnormal VF (OR 6.40; 95% CI: 3.15–12.99; *P* < 0.0001), and presence of smoking (OR 1.89, 95% CI: 1.05–3.41; *P* = 0.034).

## 4. Discussion

Understanding glaucoma risk factors can aid diagnosis and influence management decisions, including when to treat, aggressiveness of treatment, and potentially developing new treatment strategies. Our understanding of risk factors continues to evolve as the literature increases; however, publications have occasionally been conflicting thus underlining the importance of continued studies. Some confusion may relate to different study populations; for example, risk factors for the conversion of OH to OAG may differ from those for incident OAG. Similarly, risk factors for progression of OAG as well as for stages of glaucoma, and for geographic or ethnic groups, may vary. Our study was designed to describe the distribution of glaucoma risk factors in subjects with newly diagnosed treatment-naïve OH and OAG. A comparison between OH and OAG groups provided OR for each factor. Finally, risk factors were further analysed to determine an association with disease severity.

We found that optic disc characteristics such as increased C/D ratio, localized notching or thinning, NFLD, peripapillary atrophy, pallor, and disc hemorrhage were more common in OAG than OH. VF features such as increased MD, PSD, and GHT outside normal limits, defects within 10 degrees of fixation, and false negative rate were all greater in OAG. These results are expected as optic disc, and VF features are used to diagnose disease and likely should not be considered as risk factors per se. However, since most of these ocular variables lack a clear distinction between definitive glaucoma and normal (e.g., there is no cutoff for C/D ratio or MD to diagnose glaucoma), these were included in the analysis. 

A total of 11.6% (13/112) of the OH subjects' VFs were abnormal with 13% having a GHT outside normal limits and 24.1% with a defect within 10 degrees of fixation. These abnormal VFs were not felt to be glaucomatous and may represent artifact or other pathology such as age-related macular changes, and these subjects were not classified as having OAG. Other ocular features more common in OAG were worse acuity, presence of a RAPD, thinner CCT, and myopia. In the final multiple regression analysis, the only ocular variables that remained significant included increased C/D ratio, worse MD, abnormal VF, and localized notch or thinning. 

Thinner CCT has previously been considered a risk factor for both conversion of OH to OAG [2] and for the presence of OAG [40–43]. In this study OAG had significantly thinner CCT compared to OH (554.2 ± 38.2 *μ*m versus 583.4 ± 34.6 *μ*m, resp.), *P* < 0.0001; in the univariate analysis, however, CCT was not significant in the regression model.

IOP has consistently been recognized as a glaucoma risk factor including both the mean level [2, 12, 14, 21, 30, 44–47] and degree of fluctuation [33, 47]. In our study, mean IOP was higher in OH as compared to OAG, 23.4 ± 3.3 mmHg versus 20.5 ± 5.2 mmHg, respectively (*P* < 0.0001). This difference is explained both by the definition of OH (at least one IOP measurement of ≥24 mmHg) and the frequency of normal-tension glaucoma (65/292, 22.3%) in OAG. 

Evaluation of nonocular variables found the following significant differences between the groups: younger age (56.5 ± 12.6 versus 63.0 ± 13.0, *P* < 0.0001), greater proportion of Caucasian subjects (94.7% versus 82.5%. *P* = 0.013), higher weight (80.9 ± 19.6 versus 74.8 ± 15.6 kg, *P* = 0.004), greater BMI (28.6 ± 6.0 versus 26.5 ± 5.0 kg/m^2^, *P* = 0.001), and higher frequency of smoking history (46.9% versus 29.5%, *P* = 0.001) in the OH versus OAG group, respectively. Of these, only age and smoking were significant in the final multiple regression analysis.

Age is a recognized risk factor for glaucoma [48–51]. The results for smoking are controversial. We found that a history of smoking (past or current) was protective with an odds ratio of 0.36 (*P* = 0.010) for OAG versus OH. Cigarette smoking is linked to many diseases including cataracts [52] and macular degeneration [53]. Epidemiological studies have also found a negative correlation between smoking and the development of neurodegenerative disorders such as Parkinson's, and in some studies, Alzheimer's disease [54]. One large prevalence survey found no association between smoking and POAG [55]. Finally, for concomitant medications it has previously been suggested that statin use may be protective for OAG [31]. Statin use in our study was not significant in the univariate or final multiple regression analysis. 

Study location was significant in both the univariate and final multiple regression model when comparing Quebec/Atlantic region to Ontario but was not significant for comparisons including the Western region. The diagnosis of OH occurred more frequently in Quebec/Atlantic region than Ontario (48.7% versus 30.1%, resp.), and the diagnosis of OAG was more frequent in Ontario than Quebec/Atlantic region (40.4% versus 33.2%, resp.), *P* = 0.015. In the final multiple regression model, the OR of OAG versus OH was 0.27 (95% CI 0.11–0.63) for Quebec/Atlantic region versus Ontario. The significance of this finding is unclear, and further study of this is warranted.

We evaluated OAG by severity of disease at diagnosis using the definition of Damji et al. [39]. Almost half the subjects with OAG presented with moderate or advanced disease at diagnosis. Evaluation of various risk factors using logistic regression analysis found the presence of a NFLD, optic disc pallor, peripapillary atrophy, localized notch or rim thinning, and smoking to be significant in addition to variables associated with the definition of severity (MD and abnormal VF). It is interesting that smoking was a risk factor for OH when compared to OAG; however, within the OAG group, a history of smoking was a significant risk factor for advanced disease. This seemingly conflicting result deserves further study. Possibly our definition of smoking, with “yes” defined as a previous or current history of smoking, is obscuring the results. Perhaps a definition of smoking including duration and number of cigarettes per day might provide a different conclusion.

There are limitations to our study. Firstly, many of the ocular variables studied are used in the definition of OAG and likely should not be considered risk factors per se; however, these are often reported, and given the lack of a clear distinction between disease and no disease for most of these variables, we included them in the analysis. Secondly, self-reported risk factors including family and medical history may be unreliable. Finally, there may be selection bias as this study relied on the diagnosis of OAG and OH from the study site. To minimize this optic disc, visual field reading centers were responsible for independently verifying results.

## 5. Conclusions

In summary, we evaluated the distribution of previously published risk factors for glaucoma in subjects with newly diagnosed OH or OAG. In addition, within OAG, we evaluated risk factors in terms of disease severity. Logistic regression analysis determined several factors to be significantly associated with OH or OAG and severity of OAG. An interesting result was a history of smoking, which seemed to be protective for OAG versus OH; however, within OAG, it was a significant risk factor for advanced disease at presentation. In addition, subjects with advanced versus early or moderate OAG, the presence of a NFLD, optic disc pallor, peripapillary atrophy, and localized notch or rim thinning were all associated with advanced disease (OR 2.60, 5.17, 2.66, 2.08, resp.). Finally, we found that nearly 50% of subjects with OAG presented with moderate or advanced disease at initial diagnosis. This finding alone supports the importance of further studies regarding risk factors to assist in earlier diagnosis of glaucoma.

## Figures and Tables

**Table 1:** Demographic characteristics.

Characteristic	OAG (*n* = 292)	OH (*n* = 113)	*P*-value^a^
Age groups, *n* (%)			0.001
18–44 years	22 (7.5)	15 (13.3)	
45–64 years	137 (46.9)	70 (61.9)	
≥65 years	133 (45.5)	28 (24.8)	

Age, year, Mean ± SD	63.0 ± 13.0	56.5 ± 12.6	<0.0001
(Range)	(22–89)	(21–91)	

Male/female, *n* (%)	145 (49.7)/147 (50.3)	59 (52.2)/54 (47.8)	0.645

Race, *n* (%)			0.013
Caucasian	241 (82.5)	107 (94.7)	
Black	18 (6.2)	1 (0.9)	
Asian	26 (8.9)	3 (2.7)	
Other	7 (2.4)	2 (1.8)	

Weight, mean ± SD (kg)	74.8 ± 15.6	80.9 ± 19.6	0.004

Height, mean ± SD (cm)	168.0 ± 10.6	167.9 ± 9.1	0.937

BMI, Mean ± SD (kg/m^2^)	26.5 ± 5.0	28.6 ± 6.0	0.001

Location, *n* (%)			0.015
Ontario	118 (40.4)	34 (30.1)	
Quebec/atlantic	97 (33.2)	55 (48.7)	
Western	77 (26.4)	24 (21.2)	

Abbreviations: BMI: body mass index; *n*: number of subjects; OAG: open-angle glaucoma; OH: ocular hypertension; SD: standard deviation.

^a^Significance of differences between the OAG and OH groups was tested using the chi-square test for categorical data and the *t-*test for continuous data.

**Table 2:** Summary of ocular examination findings.

Variable	OAG	OH	Mean Difference^a^	*P*-value^b^
	*N* = 292	*N* = 113	(95% CI)	
Mean Intraocular pressure ±SD, mmHg^c^	20.5 ± 5.2	23.4 ± 3.3	−2.9 (−3.7, −2.0)	<0.0001
Mean central corneal thickness ±SD, microns^d^	554.2 ± 38.2	583.4 ± 34.6	−29.1 (−36.9, −21.4)	<0.0001
Mean visual acuity ±SD, LogMAR	0.2 ± 0.2	0.1 ± 0.2	0.1 (0.0, 0.1)	<0.001
Mean spherical equivalence ± SD, diopters^e^	−1.3 ± 3.3	−0.6 ± 2.5	−0.7 (−1.3, −0.0)	0.038
Abnormal anterior segment examination, *n* (%)^f^	19 (6.5)	10 (8.9)	0.7 (0.3, 1.6)	0.404
Presence of relative afferent pupillary defect, *n* (%)^f^	18 (6.2)	0 (0.0)	Infinite (Infinite)	0.005
Myopia, *n* (%)^g^	135 (46.2)	46 (40.7)	1.3 (0.8, 1.9)	0.316

Abbreviations: CI: confidence interval; LogMAR: logarithm of the minimum angle of resolution; *n*: number of subjects; OAG: open-angle glaucoma; OH: ocular hypertension; SD: standard deviation.

^a^Mean difference calculated as OAG−OH.

^b^Significance of differences between the OAG and OH groups was tested using the chi-square test for categorical data and the *t-*test for continuous data. Fisher's exact test was used for categorical data when the expected marginal size was <5.

^c^Missing data for 1 subject in the OH group.

^d^Missing data for 1 subject in the OAG group.

^e^ *n* = 266 in the OAG and *n* = 100 in the OH group.

^f^Missing data for 1 subject in both the OAG and OH groups.

^g^Data concerning myopia were collected under medical history.

**Table 3:** Summary of optic nerve head examination findings.

Variable	OAG	OH	Mean difference^a^	*P*-value^b^
	*N* = 292	*N* = 113	(95% CI)	
Mean C/D ratio^c^ ± SD	0.7 ± 0.2	0.4 ± 0.2	0.3 (0.3, 0.3)	<0.0001
Localized notch or thinning of the neuroretinal rim, *n* (%)^d^	191 (65.4)	6 (5.4)	33.3 (14.2, 78.7)	<0.0001
Presence of nerve fiber layer defect, *n* (%)^d^	55 (18.8)	0 (0.0)	Infinite (Infinite)	<0.0001
Presence of peripapillary atrophy, *n* (%)^d^	115 (39.4)	17 (15.2)	3.6 (2.1, 6.4)	<0.0001
Presence of optic disc hemorrhage, *n* (%)^d^	15 (5.1)	1 (0.9)	6.0 (0.8, 46.1)	0.082
Diffuse or localized area of pallor, *n* (%)^d^	17 (5.8)	0 (0.0)	Infinite (Infinite)	0.005
Large disc size, *n* (%)	40 (13.7)	7 (6.2)	2.4 (1.0, 5.5)	0.035

Abbreviations: C/D: cup-to-disc; CI: confidence interval; *n*: number of subjects; OAG: open-angle glaucoma; OH: ocular hypertension; SD: standard deviation.

^a^Mean differences calculated as OAG-OH.

^b^Significance of differences between the OAG and OH groups was tested using the chi-square test for categorical data and the *t-*test for continuous data. Fisher's exact test was used for categorical data when the expected marginal size was <5.

^c^C/D ratio: mean of horizontal and vertical C/D ratios; if 1 of 2 was missing, the nonmissing value was used as the average.

^d^Missing data for 1 subject in the OH group.

**Table 4:** Summary of the Humphrey visual field test findings.

Variable	OAG	OH	Mean difference^a^	*P*-value^b^
	*N* = 292	*N* = 113	(95% CI)	
Mean fixation losses ± SD^c^	9.3 ± 13.2	7.0 ± 9.6	2.3 (−0.1, 4.6)	0.058
Mean false positive rate ± SD	3.6 ± 5.9	4.3 ± 6.3	−0.71 (−2.1, 0.6)	0.299
Mean false negative rate ± SD	4.6 ± 6.7	1.9 ± 4.1	2.7 (1.6, 3.8)	<0.0001
Mean of mean deviation ± SD	−4.6 ± 5.7	−0.4 ± 1.6	−4.1 (−4.8, −3.4)	<0.0001
Mean pattern standard deviation ± SD	4.4 ± 3.6	1.7 ± 0.8	2.7 (2.3, 3.1)	<0.0001
Visual field unreliable, *n* (%)	43 (14.7)	16 (14.2)	1.0 (0.6, 1.9)	0.885
Presence of artifacts, *n* (%)^c^	10 (3.4)	5 (4.5)	0.8 (0.3, 2.3)	0.625
Abnormal visual field overall, *n* (%)^c^	181 (62.2)	13 (11.6)	12.5 (6.7, 23.4)	<0.0001
Within 10° of fixation, *n* (%)^d^	134 (45.9)	27 (24.1)	2.7 (1.6, 4.4)	<0.0001
Glaucoma hemifield test outside normal limits, *n* (%)^e^	166 (59.1)	14 (13.0)	9.7 (5.3, 17.8)	<0.0001

Abbreviations: CI: confidence interval; *n*: number of subjects; OAG: open-angle glaucoma; OH: ocular hypertension; SD: standard deviation.

^a^Mean difference calculated as OAG − OH.

^b^Significance of differences between the OAG and OH groups was tested using the chi-square test for categorical data and the *t-*test for continuous data. Fisher's exact test was used for categorical data when the expected marginal size was <5.

^c^Missing data for 1 subject in both the OAG and OH groups.

^d^Missing data for 1 subject in the OH group.

^e^ *n*: 281 in the OAG and *n*: 108 in the OH group.

**Table 5:** Summary of medical history, *n* (%).

Medical history (past or current)	OAG	OH	Odds ratio	*P*-value^a^
	*N* = 292	*N* = 113	(95% CI)	
Vascular dysfunction	128 (43.8)	37 (32.7)	1.6 (1.0, 2.5)	0.042
Hypercholestero- lemia	93 (31.8)	31 (27.4)	1.2 (0.8, 2.0)	0.387
Smoking	86 (29.5)	53 (46.9)	0.5 (0.3, 0.7)	0.001
Migraine	37 (12.7)	16 (14.2)	0.9 (0.5, 1.7)	0.690
Thyroid disease	34 (11.6)	15 (13.3)	0.9 (0.4, 1.7)	0.652
Diabetes mellitus	32 (11.0)	17 (15.0)	0.7 (0.4, 1.3)	0.258
Pseudoexfoliation syndrome	19 (6.5)	3 (2.7)	2.6 (0.7, 8.8)	0.148
Raynaud's disease	15 (5.1)	5 (4.4)	1.2 (0.4, 3.3)	0.767
Pigment dispersion syndrome	12 (4.1)	6 (5.3)	0.8 (0.3, 2.1)	0.599
Sleep apnea	9 (3.1)	7 (6.2)	0.5 (0.2, 1.3)	0.149

Abbreviations: CI = confidence interval; *n* = number of subjects; OAG = open-angle glaucoma; OH = ocular hypertension.

^a^Significance of differences between the OAG and OH groups was tested using the chi-square test; Fisher's exact test was used when the expected marginal size was <5.
